# Stereocilium height changes can account for the calcium dependence of the outer-hair-cell bundle’s resting state

**DOI:** 10.1371/journal.pone.0314728

**Published:** 2025-05-23

**Authors:** Rayan Chatterjee, Dáibhid Ó Maoiléidigh

**Affiliations:** Department of Otolaryngology–Head & Neck Surgery, Stanford University School of Medicine, Stanford, CA, USA; Astellas, UNITED STATES OF AMERICA

## Abstract

Outer-hair-cell bundles are sensory organelles required for normal hearing in mammals. These bundles convert sound-induced forces into receptor currents. This conversion depends on the resting receptor current of each bundle, which increases when extracellular calcium is decreased to the physiological level. How extracellular calcium regulates the bundle’s resting state is not well understood. We propose a mechanism explaining how extracellular calcium can regulate the outer-hair-cell bundle’s resting state. Each bundle comprises filamentous stereocilia linked by gating springs that are attached to ion channels. Sound-induced forces deflect stereocilia, increasing and decreasing gating-spring tensions, opening and closing the ion channels, resulting in an oscillating receptor current. We hypothesize that decreasing extracellular calcium, decreases the heights of the shorter stereocilia, increasing resting gating-spring tensions, which increases the resting receptor current and decreases the bundle’s resting deflection. To determine the plausibility of this mechanism, we build a mathematical model of an outer-hair-cell bundle and calibrate the model using seven independent experimental observations. The calibrated model shows that the mechanism is quantitatively plausible and predicts that a decrease of only 10 nm in the heights of the shorter stereocilia when extracellular calcium is lowered is sufficient to explain the observed increase in the resting receptor current. The model predicts the values of nine parameters and makes several additional predictions.

## Introduction

In our ears, outer-hair-cell bundles (OHBs) convert sound-induced forces into receptor currents [1,2]. These receptor currents drive a process known as the cochlear amplifier, which is responsible of our hearing’s high sensitivity, sharp frequency selectivity, and wide dynamic range. Although OHBs are required for normal hearing, we do not fully understand how they work. Experiments show that the OHB’s resting receptor current (no stimulus forces) increases when the extracellular calcium concentration is lowered to physiological levels [3–6]. We propose a mechanism that can account for changes in an OHB’s resting state owing to changes in extracellular calcium.

**Fig 1:**
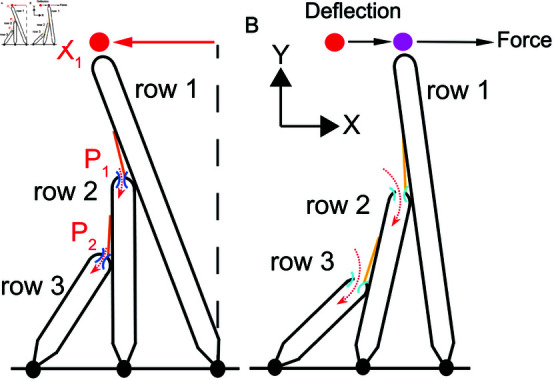
OHB morphology and response to stimulation. (A) A schematic of an OHB column is shown. Because the OHB model comprises 28 identical and independent columns, it is represented using a single column. An OHB consists of three rows of stereocilia decreasing in height from row 1 (tallest) to row 3 (shortest). Gating springs (orange) link each stereocilium with its shorter neighbor. The stereocilia in row 2 and row 3 have ion channels (blue) at their tips. The displacement of the OHB (*X*_1_) equals the displacement of the row-1 tip relative to the hair-cell apex perpendicular (vertical dashed line). The normalized receptor current equals the normalized sum of the normalized channel currents *P*_1_ and *P*_2_ ($P=(P_{1}+P_{2})/2$). (B) A schematic of the OHB responding to a stimulus force is shown. When an OHB is deflected towards row 1 (purple colored dot) in response to a stimulus, the gating springs extend (light orange) and the channels open (light blue), increasing the ionic currents into the OHB (dashed red arrows).

An OHB comprises filaments, known as stereocilia, emanating from the outer hair cell’s apical surface (Fig 1) [1,2]. Within an OHB, stereocilia of similar height form rows and stereocilia of differing height form columns. In a column, stereocilia from different rows are linked by gating springs, made of proteinaceous tip links and other elements in series with the tip links. At the lower ends of the gating springs sit ion channels, embedded in the shorter stereocilia of rows 2 and 3. Stimulus forces toward the tallest row (row 1) deflect the stereocilia, which pivot at their insertion points, increase gating-spring tensions, and open the ion channels, through which the receptor current flows. The receptor-current response to stimulation depends on the resting current, which in turn depends on the extracellular calcium concentration [3–6].

According to most prior work, the extracellular calcium concentration is low (≤ 50 μM) under normal physiological conditions [3,4,7–11]. In low extracellular calcium, the resting current is about half the maximum receptor current and is a major component of the inner ear’s silent current [3,4,11,12]. However, in higher calcium concentration environments (≥ 0.5 mM), the resting current is smaller (< 0.2 of the maximum receptor current) [3,4,11]. In other words, decreasing extracellular calcium opens the ion channels. Decreasing the extracellular calcium concentration also decreases the heights of the shorter stereocilia, owing to the change in calcium entering through the open ion channels at rest [13]. We propose that lowering calcium increases the normalized resting current by decreasing the heights of the shorter stereocilia. Throughout the paper, we focus on the normalized receptor current, which equals the receptor current relative to the maximum current.

Here, we quantitatively evaluate the plausibility of our proposal. We build a mathematical model of the OHB, based on seven morphological measurements and calibrated using seven physiological measurements. The calibration procedure predicts the values of nine OHB parameters. Using the model, we show that decreasing stereocilium heights in low calcium can explain the increase in the resting current. The mathematical model also predicts that the resting OHB displacement and resting gating-spring tensions change when the calcium concentration is changed.

## Methods

The OHB mathematical model comprises 28 independent and identical columns of stereocilia. Each column contains three stereocilia of differing height linked by gating springs (Fig 1). The stereocilia remain in sliding contact with their nearest neighbors in each column when they pivot [14]. Consequently, the displacements of the shorter stereocilia (rows 2 and 3) and the gating lengths (gating length equals the stereocilium radius plus the tip-link length) are dictated by the displacement of the tallest row of stereocilia (row 1) and the morphology of the OHB (Fig S1 and Table 1). The stereocilia pivot in 2D and have the same pivot stiffnesses and unloaded states. Likewise, the gating springs have same stiffnesses and unloaded lengths. The resting gating length equals the stereocilium radius plus the measured resting tip-link length [15].

**Table 1:** Morphological parameters for the OHB model. Values are given as mean ± standard deviation and citations to the data sources are given in parentheses.

Parameter	Value	Calcium concentration
Row-1 height ($\ell_{1}$)	4100 ± 632 nm [16,17]	1.5–2 mM
Row-2 height ($\ell_{2}$)	2100 ± 700 nm [16,17]	1.5–2 mM
Row-3 height ($\ell_{3}$)	1300 ± 400 nm [16,17]	1.5–2 mM
Number of columns (*N*_*c*_)	28 [18]	2 mM
Stereocilium width (*c*)	289 ± 82 nm [16,17]	1.5–2 mM
Row 1-2 spacing (*a*_1_)	588 ± 43 nm [18]	2 mM
Row 2-3 spacing (*a*_2_)	618 ± 83 nm [18]	2 mM

Each gating spring is attached to a mechanoelectrical-transduction channel with two states, open and closed [19]. The current through the channel normalized by the maximum current equals the open probability of the channel, which depends on the gating length. Opening a channel decreases the gating-spring length by an amount known as the gating swing [20]. The gating-spring length equals the gating length minus the product of the gating swing and the channel open probability. The receptor current equals the sum of the currents through rows 2 and 3. We normalize the receptor current using the maximum receptor current. In other words, the model OHB fits and predicts the normalized receptor current, but does not specify the absolute receptor current.

The number of stereocilia, the spacing between the stereocilium pivot points, the widths of the stereocilia, and the heights of the stereocilia are based on published experimental observations on OHBs from the 4-kHz characteristic-frequency region in rats (Table 1) [16–18]. Using published experimental observations, we create constraint equations that enable us to derive nine additional parameter values describing the OHB model. This OHB model and the fitting procedure are described in detail in the S1 Text.

Mathematica 13 was used to solve the mathematical model (S2 Code). Data from Johnson et. al (2011) was extracted using WebPlotDigitizer (automeris.io) and fitted using Mathematica 13 (S3 Data–S6 Data) [4].

## Results

To determine how the resting state of the OHB depends on extracellular calcium, we build a model of the OHB constrained by published experimental observations. The morphology of the OHB model (stereocilium number, heights, widths, and pivot separations) is based on published experimental observations (Table 1) [16–18]. The OHB is described by identical columns of three stereocilia and these stereocilia remain in sliding contact. These choices greatly reduce the number of parameters and variables needed to describe the OHB, which enables us to calculate the values of all the remaining OHB parameters (Methods and S1 Text).

Because we lack many OHB morphology measurements in low calcium, we assume that most of the missing morphology data in low calcium are the same as prior observations of the OHB’s morphology in high calcium (Table 1) [16–18]. However, we allow row-2 heights to differ and row-3 heights to differ in low and high calcium. We assume that the height differences in low and high calcium are small and approximate the low-calcium heights using data in high calcium (Table 1) [16–18]. Then, we calculate the resting heights of rows 2 and 3 in high extracellular calcium by fitting the model OHB to prior physiological measurements (Table 2). In other words, we do not assume a mechanism driving the height changes, a timescale for the height changes, or a specific calcium concentration (calcium concentration is not a parameter in the model OHB) and only assume that the resting heights differ in low and high extracellular calcium. We show below that the heights of rows 2 and 3 depend little on the external calcium concentration in the model OHB but that these small changes have large effects on the resting state, and that the model’s conclusions do not depend strongly on the absolute resting heights.

**Table 2:** Emergent property values (mean ± standard deviation). Seven independent experimental observations are listed. The resting current in high calcium ($P_{P}^{*}$) is determined by the measured activation curve width ($2\Delta_{J}$) and center (*X*_*J*_).

Emergent property	Experiment	Model
**Low calcium**		
Resting current ($P_{E}^{*}$)	0.5 [4,16]	0.5
Resting tip-link length ($x_{TL}^{*}$)	186 ± 38 nm [15]	186 nm
OHB stiffness without gating springs ($K_{HB}^{noGS}$)	5.5 ± 2 mN/m [16]	5.5 mN/m
Deflection from breaking gating springs ($\Delta_{E}$)	45 ± 10 nm [16]	45 nm
**High calcium**		
Activation-curve width (0.27–0.73) ($2\Delta_{J}$)	34 nm [4]	34 nm
Activation-curve center relative to rest (*X*_*J*_)	40 nm [4]	40 nm
Resting current ($P_{P}^{*}$)	$0.087=(1+\exp(X_{J}/\Delta_{J}))-1$ [4]	0.087
OHB stiffness with gating springs (*K*_*HB*_)	8.6 ± 2.3 mN/m [16]	8.6 mN/m

We define the X direction to be parallel to the hair-cell apex and the Y direction to be perpendicular to the hair-cell apex (Fig 1). Displacements of the stereocilium tips in the X direction and in the direction of increasing stereocilium height are positive. In most experiments, the X displacement is measured relative to the resting displacement of the OHB, but the resting displacement of the OHB is not determined [3–5,16]. Here, we define the X displacement to be relative to the hair-cell apex perpendicular and calculate the resting displacement of the OHB (Fig 1).

### The OHB model quantitatively accounts for seven independent experimental observations

**Fig 2:**
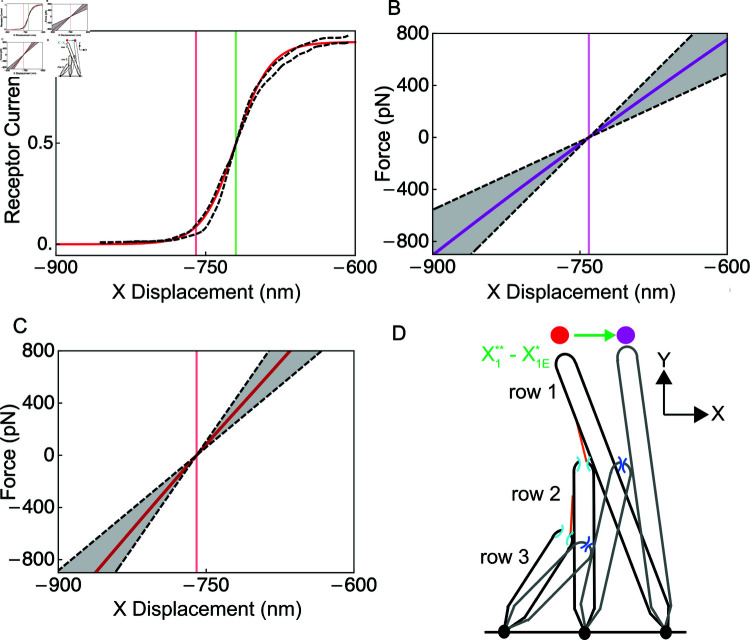
The OHB model fits published experimental observations. (A) The receptor current is shown versus the OHB displacement (known as the activation curve) for the model (red line) and for experiment (black dashed lines). The model agrees with the average of the two experimental activation curves. The extracellular calcium concentration is high. Vertical lines indicate OHB displacement at rest (red) and at half-activation (green) for the model. (B) The force applied to the OHB versus its displacement is shown for the model (purple line) and for experiment (black dashed lines indicate the mean ± the standard deviation) when the OHB has no gating springs. The vertical purple line indicates the resting OHB displacement for the model. (C) The force applied to the OHB versus its displacement is shown for the model (red line) and for experiment (black dashed lines indicate the mean ± the standard deviation) when the OHB has gating springs. The vertical red line indicates the resting OHB displacement for the model. (D) Schematic representations of the OHB are shown with gating springs (black) and without gating springs (grey), illustrating that the resting OHB displacement increases (green arrow, $X_{1}^{**}-X_{1E}^{*}$) when the gating springs are broken. The dots indicate the resting displacement with (red) and without (purple) gating springs.

In response to a stimulus toward row 1, the stereocilia are displaced in the positive direction and the mechanoelectrical-transduction channels open. To reduce the number of parameters, the model OHB produces the receptor current normalized by the maximum receptor current rather than the absolute receptor current. The model OHB reproduces the measured normalized receptor-current dependence on the OHB’s displacement (the tip displacement of the row-1 stereocilium), including the resting receptor current in high calcium (Fig 2A and Table 2). When the gating springs are cut in experiment, the OHB’s stiffness (slope of the force versus displacement curve) decreases (Fig 2B,C and Table 2). The model OHB captures the measured stiffnesses of the OHB with (in high calcium) and without gating-springs. For low calcium, the resting length of the tip link and the resting receptor current in the OHB model equal the values measured experimentally (Table 2). When the gating springs are cut in low calcium, the resting displacement of the OHB increases in experiment, a change which is also matched by the OHB model (Fig 2B–D and Table 2). Overall, the model OHB quantitatively fits seven independent experimental observations.

### The OHB model predicts the values of nine parameters

**Fig 3:**
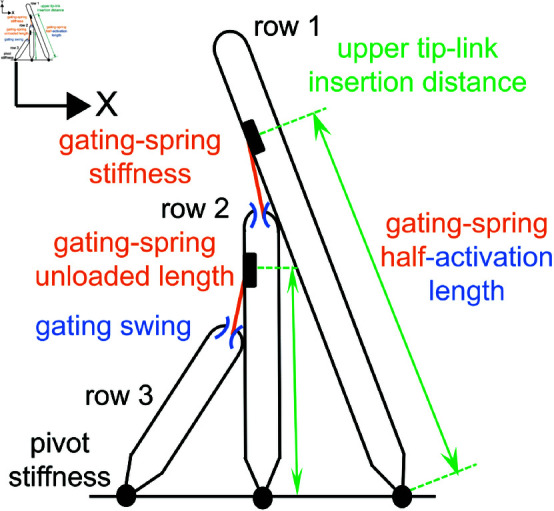
The OHB model predicts the values of several OHB properties. A schematic representing the OHB is shown highlighting the parameter values predicted using the OHB model (values are summarized in Table 3). These parameters are the upper tip-link insertion distances for row 1 and row 2 (green arrows; *b*_1_ and *b*_2_), the stiffness of the stereocilium pivots (black dots; κ), the stiffness (*k*_*gs*_) and the unloaded length (*x*_*u*_) of the gating springs (orange), the gating swing of the channels (blue; *d*), and the half-activation length of the gating springs (orange/blue; the half-activation length depends on the gating springs and the channels; *x*_*h*_).

The model OHB predicts the values of 17 unknowns (9 parameter values and 8 state values) based on 17 constraints and prior experimental observations (Fig 3, Tables 1–3,S1 Text, Table S1 in S1 Text). Following prior work, we assume that myosin motors set the resting gating-spring lengths to be the same [18]. Then, based on the measured resting receptor current of 0.5 in low calcium, the resting currents are 0.5 in low calcium for all the mechanoelectrical-transduction channels. The sliding contact assumption enables us to find the resting displacement of the OHB when its gating springs are cut and then to find the resting displacement of the OHB with intact gating springs (–786 nm) from the measured displacement of the OHB caused by cutting the gating springs. The resting displacements are negative relative to the hair-cell apex perpendicular owing to the balance of forces and torques at rest.

**Table 3:** Predicted parameter values and resting-state variable values. The parameters are the upper tip-link insertion distances for row 1 and row 2 (*b*_1_ and *b*_2_), the stiffness of the stereocilium pivots (κ), the stiffness (*k*_*gs*_) and the unloaded length (*x*_*u*_) of the gating springs, the gating swing of the channels (d), the half-activation length of the gating springs (*x*_*h*_), and the heights of row 2 and row 3 in high calcium ($\ell_{2P}$ and $\ell_{3P}$). The resting-state variables are the resting OHB displacement without gating springs ($X_{1}^{**}$), the resting OHB displacement in low calcium ($X_{1E}^{*}$), the resting gating lengths in low calcium ($x_{1}(X_{1E}^{*})$ and $x_{2}(X_{1E}^{*})$), the resting tip-link length plus the stereocilium radius in low calcium ($x_{E}^{*}$), the resting OHB displacement in high calcium ($X_{1P}^{*}$), and the resting gating lengths in high calcium ($x_{1}(\ell_{2P},X_{1P}^{*})$ and $x_{2}(\ell_{2P},\ell_{3P},X_{1P}^{*})$).

Unknown	Prediction	Unknown	Prediction
**Low calcium**		**High calcium**	
**Parameter**	**Value**	**Parameter**	**Value**
κ	1.0 fN.m/rad	$\ell_{2P}$	2110.0 nm
*b*_1_	2490.1 nm	$\ell_{3P}$	1309.7 nm
*b*_2_	1589.3 nm	*k*_*gs*_	2.9 mN/m
*x*_*h*_	330.5 nm	*d*	0.6 nm
*x*_*u*_	319.1 nm		
**Variable**	**Value**	**Variable**	**Value**
$X_{1}^{**}$	–741.0 nm	$X_{1P}^{*}$	–759.8 nm
$X_{1E}^{*}$	–786.0 nm	$x_{1}(\ell_{2P},X_{1P}^{*})$	324.9 nm
$x_{1}(X_{1E}^{*})$	330.5 nm	$x_{2}(\ell_{2P},\ell_{3P},X_{1P}^{*})$	324.9 nm
$x_{2}(X_{1E}^{*})$	330.5 nm		
$x_{E}^{*}$	330.5 nm		

Combining the resting displacement of the intact OHB with its geometry yields the resting sliding contact points. In the model OHB, the resting gating lengths equal the stereocilium radius plus the measured resting length of the tip links. We calculate the upper tip-link attachment locations using the resting gating lengths and the resting sliding contact points. The gating length required for the channel current to be 0.5 (the half-activation length) equals the resting gating lengths in low calcium. Using the measured stiffness of the OHB after its gating spring have been cut, we find a pivot stiffness value of 1.0 fN.m/rad (angular stiffness), which is smaller than prior work using an identical-gating model (1.3 fN.m/rad), because the prior model assumes stereocilia in different rows contribute equally to OHB stiffness and we use more stereocilia (84 here versus 70.6 in prior work) [16].

Using the calculated values of the upper tip-link attachment points, the half-activation length, and the pivot stiffness we find many additional parameters (Table 3). We find the gating-spring stiffness (2.9 mN/m), the unloaded length of the gating spring (319 nm), the gating swing (0.6 nm), and the heights of the row 2 (2110 nm) and 3 (1310 nm) stereocilia in high calcium (Table 3). To find these parameters, we use several prior measurements, including the measured stiffness of the OHB in high calcium, the measured OHB activation curve in high calcium, and the measured displacement of the OHB caused by cutting the gating springs in low calcium (Table 2) [4,16]. The predicted value of the gating-spring stiffness (2.9 mN/m) is smaller than the value found using an identical-gating model (3.7 mN/m) in prior work, because the identical-gating model assumes that all gating springs contribute equally to OHB stiffness, we use fewer gating springs (56 here versus 65 in prior work), and we use larger pivot spacings (603 nm on average here versus 462 nm in prior work) [16]. In contrast, the predicted value of the gating swing equals the value assumed in a prior OHB model [21].

### Decreasing the heights of rows 2 and 3 explains the change in resting state from high to low calcium

**Fig 4:**
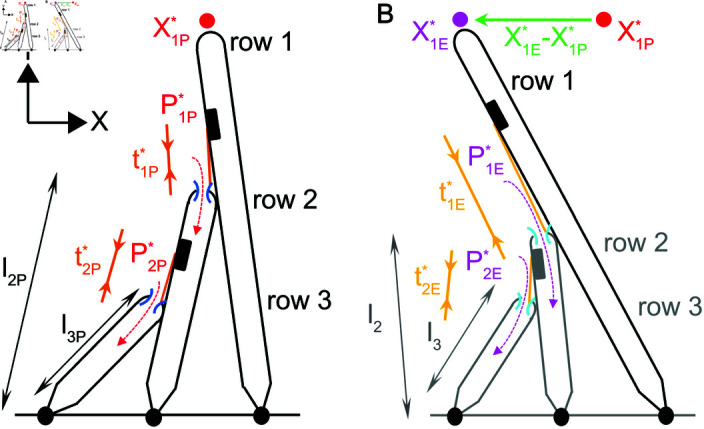
A decrease in the row-2 and row-3 heights in low extracellular calcium is predicted by the model OHB to increase the resting receptor current and decrease the resting deflection of the OHB. (A) A schematic representing the OHB in high extracellular calcium is shown. The resting current (red-dashed arrows; $P_{1P}^{*}$ and $P_{2P}^{*}$), resting channels (dark-blue arcs), resting deflection in the X direction (red dot; $X_{1P}^{*}$), resting tensions (dark-orange arrows; $t_{1P}^{*}$ and $t_{2P}^{*}$), and the heights of rows 2 and 3 (black arrows; $\ell_{2P}$ and $\ell_{3P}$) are indicated. (B) A schematic representing the OHB in low extracellular calcium is shown. The resting current (purple-dashed arrows, predicted to increase relative to high extracellular calcium; $P_{1E}^{*}$ and $P_{2E}^{*}$), resting channels (light-blue arcs, predicted to be more open relative to high extracellular calcium), resting deflection in the X direction (purple dot, the green arrow indicates the predicted decrease relative to high extracellular calcium; $X_{1E}^{*}$), resting tensions (light-orange arrows, predicted to increase relative to high extracellular calcium; $t_{1E}^{*}$ and $t_{2E}^{*}$), and the row-2 and row-3 heights (grey, predicted to decrease relative to high extracellular calcium; $\ell_{2}$ and $\ell_{3}$) are indicated.

To account for the measured differences in resting receptor current (0.087 in high calcium versus 0.5 in low calcium), we allow the heights of row 2 and 3 to differ in high and low calcium in the model OHB (Fig 4). The OHB model predicts that a decrease in height of about 10 nm in low calcium increases the resting tensions (16.6 pN in high calcium to 31.9 pN in low calcium) and decreases the resting displacement of the OHB (–760 nm in high calcium to –786 nm in low calcium). Additionally, the OHB model predicts similar height decreases for row 2 (–10.0 nm) and row 3 (–9.7 nm) (Table 3).

**Fig 5:**
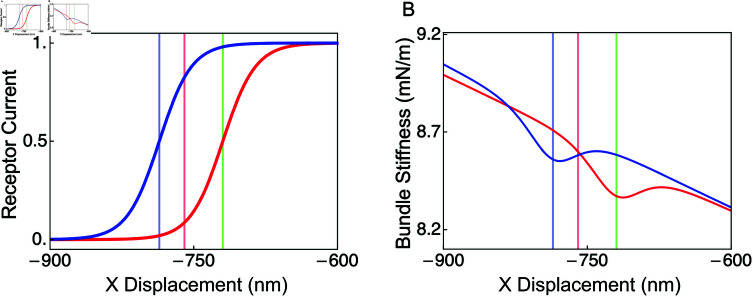
The OHB model predicts the activation curve in low extracellular calcium and the OHB stiffnesses in high and low extracellular calcium. The receptor currents (A) and the OHB stiffnesses (B) are shown versus the OHB displacement in high (red line) and low (blue line) extracellular calcium. Vertical lines indicate OHB displacements in high extracellular calcium at rest (red), in high extracellular calcium at half activation (green), and in low extracellular calcium at rest (blue).

We fit the model OHB to the receptor-current activation curve and OHB stiffness near the resting state in high calcium (Table 2,Fig 2). The model OHB predicts that the activation curve center decreases in low calcium by 66 nm (Fig 5A). Owing to the geometry of an OHB column, the model OHB also predicts that the OHB’s stiffness decreases with OHB displacement in high and low calcium (Fig 5B). In high and low calcium, mechanoelectrical-transduction channel gating decreases the OHB’s stiffness when the receptor current is near 0.5, a phenomenon known as gating compliance [20]. However, the predicted angular displacements of the stereocilia increase almost linearly and their predicted sliding contact positions decrease almost linearly with the OHB’s displacement (Figs S2 and S3 in S1 Text).

The resting angles between the tip-links and the taller stereocilia are small ($\arcsin(c/(2x_{i}(X_{1E}^{*})))$ $=26^{\circ }$ in low calcium and $\arcsin(c/(2x_{i}(\ell_{2P},X_{1P}^{*})))=26^{\circ }$ in high calcium), in agreement with prior measurements of 12–30∘ (Tables 1 and 3) [15]. Because these resting angles are small, the gating lengths increase linearly as functions of OHB displacement with slopes approximately equal to $a_{1}/\ell_{1}=0.14$ (Table 1). These slopes are known as the geometric gains [20]. The geometric gains in low calcium are 0.13 for gating length 1 and 0.15 for gating length 2, and the geometric gains in high calcium are 0.13 for gating length 1 and 0.15 for gating length 2 (Fig S4 in S1 Text). Within the physiological range of OHB displacements, the predicted row-2 and row-3 gating lengths are very similar, which make the predicted channel currents versus the OHB’s displacement almost identical (Figs S4 and S5 in S1 Text).

### The height decreases predicted by the OHB model in low extracellular calcium depend little on the absolute row heights

The measured stereocilium heights are variable, which may impact the OHB model’s predictions (Table 1) [16–18]). To assesses the impact, we refit the OHB model to the experimental data using minimum or maximum values for each stereocilium height within one standard deviation of the mean that are consistent with the other morphological parameters (code, fitting, and figures in S7 Code–S12 Code). We change each row height independently to yield six sets of height-variation results, namely, row 1 minimized, row 1 maximized, row 2 minimized, row 2 maximized, row 3 minimized, and row 3 maximized.

The height-variation models fit the experimental data equally well as the default OHB model (the emergent property values for each model are identical to those in Table 2), but yield different variable and parameter values (Tables S2–S7 in S1 Text). In the height-variation models, the resting displacements vary by 287 nm (–672 nm to –959 nm in low calcium and –646 nm to –929 nm in high calcium; Tables S2–S7 in S1 Text). Parameter values that vary include pivot stiffness (0.7 fN.m/rad to 1.4 fN.m/rad), gating-spring stiffness (2.1 mN/m to 3.8 mN/m), and gating swing (0.5 nm to 0.7 nm). From high to low calcium, row-2 and row-3 heights decrease by similar amounts in each height-variation model (0.4 nm difference at most) and only vary between –8.4 nm and –11.7 nm.

## Discussion

Our results show that shorter-row height changes owing to changes in calcium concentration is a plausible mechanism for explaining resting-state changes in OHBs. Accounting for seven different experimental observations from three different papers using a single mathematical model, suggests that the experimental observations are quantitatively consistent across these publications [4,15,16]. In addition to predicting the values of nine parameters, the mathematical model makes several other predictions.

The predicted increase of 5.6 nm in the resting gating length (equaling the resting tip-link length plus the stereocilium radius) caused by lowering the calcium concentration is consistent with the measured increase of 21±69 nm in the resting tip-link length (164.4±57.3 nm in 1 mM calcium and 185.8±37.6 nm in 50 μM calcium; mean ± standard error) [15]. This agreement implies that the stereocilium radius need not change to account for the predicted gating-length change.

When extracellular calcium is decreased, the mathematical model predicts that the resting OHB displacement decreases by 26 nm, the activation curve shifts in the negative direction, and the center of the nonlinearity associated with channel gating shifts in the negative direction (Table 3; Fig 5). The predicted 26 nm decrease in the resting OHB displacement when the calcium concentration is decreased is similar to the measured decrease in displacement of about 20 nm when the calcium concentration is decreased from 0.5 mM to a lower concentration [16]. Negative shifts in the activation curve and center of the nonlinearity relative to the resting state have been observed [3–5,22]. A prior mathematical model of receptor-current adaptation (a decrease in the receptor current during a sustained static displacement stimulus) also predicts that activation curves shift in the negative direction when extracellular calcium is lowered, suggesting that there may be several contributions to the activation curve shift [22].

We fit the model OHB to data that are, as much as possible, for the same species, for similar cochlear locations, and for similar ages [4,15,16]. However, the predictions of the model OHB apply to OHBs in general, because there are other publications reporting similar data in OHBs, including the resting current in low and high calcium, the resting tip-link length, the OHB stiffness, the OHB stiffness without gating springs, the activation-curve width, and the activation curve center relative to rest [11,22–26].

Although most publications suggest that the extracellular calcium concentration is ≤ 50 μM *in vivo* [3,4,7–11], one publication suggests that extracellular calcium concentration around the OHB is higher [27]. However, Stimbu et al. were unable to provide an estimate of the concentration [27]. The OHB is embedded in an overlying membrane, called the tectorial membrane *in vivo* [1,2]. Strimbu et al. found that the calcium concentration in the tectorial membrane above the OHBs may be >300 μM, but it is unclear how much this high tectorial-membrane calcium would increase the free extracellular calcium around the OHB.

The negative resting displacement of the OHB predicted by the model in high calcium (–760 nm; $\arcsin(-760/4100)=-11^{\circ }$, in which the OHB height is 4100 nm; Tables 1 and 3) is quantitatively consistent with prior observations of OHBs ($-8^{\circ }$ to $-12^{\circ }$ on average) [28]. We find a similar negative resting displacement in low calcium –786 nm (Table 3). *In situ* measurements in high calcium show positive resting displacements, suggesting that the tectorial membrane biases the OHB in the positive direction ($15^{\circ }$ on average at the cochlear apex) [29]. However, recent *in vivo* measurements suggest that the tectorial membrane does not bias the resting OHB displacement [11]. The resting OHB receptor current in low calcium is about 0.5 with and without the tectorial membrane, implying that the tectorial membrane does not bias the resting OHB displacement [11]. It is unlikely that receptor-current adaptation can maintain a receptor current close to 0.5 if the resting displacement were shifted from our calculated value of –786 nm to 1161 nm ($4100\times \sin(15^{\circ })$ nm; Tables 1 and 3) by the tectorial membrane, because there is little adaptation for OHB displacements larger than 500 nm [30–32]. Determining whether the resting displacement is positive or negative *in vivo* is important for our understanding of the cochlear amplifier [33,34].

Like a prior 3D mathematical model of the OHB and prior morphological observations, row 1 leans toward row 3 and row 3 leans toward row 1 in the essentially 2D model presented here (all columns are identical and independent and are described by the 2D motions of a single column) [17,18,28]. However, unlike the prior 3D mathematical model of the OHB, the 2D model produces similar channel currents in rows 2 and 3 (Fig S5) [18]. The current similarity is a consequence of the 2D and sliding-contact approximations, because a 3D OHB model lacking the sliding-contact assumption shows that row-2 and row-3 currents differ substantially [18]. Nonetheless, these approximations enable us to better understand the OHB and to fit the model exactly to the experimental data (Table 2 and 3). We expect the 2D model predictions to hold semi-quantitatively in 3D (e.g., we expect decreases in row-2 and row-3 heights by about 10 nm to account for the resting-state differences between high and low extracellular calcium in 3D), because the 3D model is an extension of the 2D model and has similar parameter values [18]. Because OHB columns are coupled in 3D, row-1 displacements toward row 3 are associated with narrowing the V-shape formed by the row-1 stereocilium tips [18]. Correspondingly, we expect decreasing extracellular calcium to displace row-1 stereocilia toward row 3, narrowing the tip V-shape.

OHB stiffness has been observed to increase when the calcium concentration is lowered from 1.5 mM to 0.02 mM [22]. We find that this change in OHB stiffness is not explained by changing the row-2 and row-3 stereocilium heights, implying that additional calcium-dependent mechanisms are needed to explain the observation (Fig 5). Possible mechanisms to explain the OHB stiffness increase when calcium decreases include, decreasing the unloaded length of the gating spring, increasing gating-spring stiffness, and increasing the stiffness of an element in series with the gating spring [22,35].

Why might extracellular calcium affect stereocilium heights? One possibility is that a decrease in extracellular calcium decreases calcium in stereocilia, decreasing the rate of actin polymerization in row-2 and row-3 stereocilia, decreasing row-2 and row-3 heights [13]. However, the rate of height changes owing to actin polymerization is limited. The polymerization rate is 1–10 monomers per second and each actin monomer is about 6 nm long [36]. To increase stereocilium heights by 10 nm would require 1.7 monomers on average, which would take 0.17–1.7 seconds.

There are several lines of experimental evidence showing that decreasing calcium in stereocilia decreases the heights of shorter stereocilia [13,37,38]. Blocking the channel, buffering intracellular calcium, or decreasing extracellular calcium from 1.8 mM to 100 μM decreases calcium in stereocilia and decreases row-2 and row-3 heights by at least 100 nm on average [13]. Although experimental measurements of height decreases owing to extracellular calcium reductions are for immature OHBs (postnatal-day 4), stereocilium cytoskeleton components turnover in the 500-nm region nearest the tips of stereocilia in mature bundles, suggesting that stereocilium height changes of 10 nm in mature OHBs are plausible [39–42]. Recent work shows that stereocilium height changes caused by blocking the mechanoelectrical-transduction channels require myosin 15A [38].

Another possible mechanism underlying height differences in different calcium environments, is that there are calcium-dependent elastic elements within stereocilia, whose resting lengths regulate the resting stereocilium heights. Decreasing extracellular calcium decreases calcium in stereocilia, which might increase the stiffness of the elements, decreasing stereocilium heights. An element within OHB stereocilia with a calcium-dependent stiffness has been proposed previously to affect the resting current, but the potential effects on stereocilium heights was not described [35]. The rate of height changes owing to this mechanism is unknown.

Several calcium-dependent process might change the resting state of the OHB [26]. To avoid overfitting, we have not investigated these additional processes here. It is possible that calcium-dependent height changes are smaller than we predict, because other calcium-dependent processes might account for some of the resting-current changes [26]. Our proposal is independent from some of these processes (e.g., lipid bilayer modulation by calcium and calcium changing permeation of the mechanoelectrical-transduction channel), but might contribute to other processes [6,26]. Like decreasing extracellular calcium, depolarization decreases intracellular calcium and increases the resting current magnitude, suggesting that depolarization might decrease row-2 and row-3 heights [5,6,13]. Depolarization affects the receptor current on timescales of 100 ms to several seconds, so an actin polymerization mechanism would be sufficiently fast to contribute to depolarization regulation of the resting current [5,6]. In contrast, slow adaptation is a calcium-dependent process that decreases the receptor current with a timescale of about 20 ms in OHBs, implying that height changes based on actin polymerization alone are not sufficiently rapid to account for slow adaptation [5,26]. However, a calcium-dependent elastic element mechanism that changes stereocilium heights might be sufficiently fast to contribute to slow adaptation [35].

Even if row-2 and row-3 height changes do not contribute to adaptation, we show that small height changes (0.5 % for row 2 and 0.7 % for row 3) have big effects on the resting current and the sensitivity of the OHB. In other words, decreasing the row-2 and row-3 heights by less then 1 % increases the resting current from 0.087 to 0.5, which dramatically increases the sensitivity of the receptor current to small stimuli (Fig 5A; slopes at resting displacements). It is likely difficult for the OHB to maintain the stereocilium heights with a precision of less then 1 % (for example, with a precision of less than two actin monomers) [36]. Adaptation is needed to compensate for height fluctuations, maintaining the operating point of the OHB to ensure our hearing’s great sensitivity to weak sounds [1].

## Conclusions

We propose a new mechanism for regulating the resting state of the OHB that brings together several different experimental observations. Decreasing extracellular calcium decreases the heights of rows 2 and 3, increasing resting gating-spring tensions, deflecting the OHB in the negative direction and increasing the resting receptor current. The mathematical model predicts the values of nine parameters and the resting values of eight variables. Some predictions agree with prior experimental data, some predictions imply that additional mechanisms are needed to explain experimental data, and all predictions can in principle be tested experimentally.

## Supporting information

S1 Text chatterjeeSI.pdf Mathematical model description and supporting figures.

S2 Code resting_ca_dependence.nb Mathematical model code (Mathematica 13.3.1).

S3 Data Jd1e1b.csv Experimental data used for fitting mathematical model.

S4 Data Jd1e2b.csv Experimental data used for fitting mathematical model.

S5 Data Johnsonsine.csv Experimental data used for fitting mathematical model.

S6 Data Johnson20111E.csv Experimental data used for fitting mathematical model.

S7 Code resting_ca_dependence_l1Min.nb Mathematical model code using a minimum value for the row-1 height consistent with experimental data (Mathematica 13.3.1).

S8 Code resting_ca_dependence_l1Max.nb Mathematical model code using a maximum value for the row-1 height consistent with experimental data (Mathematica 13.3.1).

S9 Code resting_ca_dependence_l2Min.nb Mathematical model code using a minimum value for the row-2 height consistent with experimental data (Mathematica 13.3.1).

S10 Code resting_ca_dependence_l2Max.nb Mathematical model code using a maximum value for the row-2 height consistent with experimental data (Mathematica 13.3.1).

S11 Code resting_ca_dependence_l3Min.nb Mathematical model code using a minimum value for the row-3 height consistent with experimental data (Mathematica 13.3.1).

S12 Code resting_ca_dependence_l3Max.nb Mathematical model code using a maximum value for the row-3 height consistent with experimental data (Mathematica 13.3.1).
