# Supplementary material for: Stereocilium height changes can account for the calcium dependence of the outer-hair-cell bundle’s resting state
Source: PLoS One. 2025 May 23;20(5):e0314728. doi: 10.1371/journal.pone.0314728 (PMC12101656; doi:10.1371/journal.pone.0314728)
Supplement: S1 Text — Mathematical model description and supporting figures. [file pone.0314728.s001.pdf]

Supporting information for

Stereocilium height changes can account for the calcium dependence of the outer-hair-cell bundle's resting state

Rayan Chatterjee[a] and Dáibhid Ó Maoiléidigh[a,1]

[a]Department of Otolaryngology-Head and Neck Surgery,

Stanford University, Stanford, CA, USA

[1] To whom correspondence should be addressed. Email: dmelody@stanford.edu

Mathematical model

Here we describe the OHB mathematical model and the method for fitting the model to experimental data.

Governing equation

The OHB model comprises 28 identical and independent columns consisting of three rows of stereocilia, two gating springs, and two ion channels (Fig S1). Because the columns are identical and independent, we need only describe a single column and can account for the output of the whole OHB by multiplying by the number of columns. We describe the stereocilia as massless, rigid, cylindrical rods topped by hemispheres with the same diameters (c) as the cylinders. The stereocilia pivot on the hair-cell apex and have angular displacements from the hair-cell apex perpendicular given by θ_i, in which $i \in \{1, 2, 3\}$ denotes row i. Stereocilia have the same angular stiffness (κ) and unloaded angular displacement (θ_u). The symbols x_i indicate the ith gating length between stereocilia i and $i+1$ and P_i is the normalized current (open probability) through the mechanoelectrical-transduction channel in stereocilium $i+1$, in which $i \in \{1, 2\}$. Each gating complex has the same gating-spring stiffness (k_{gs}), unloaded gating-spring length (x_u), gating length causing a normalized current of one half (x_h, half-activation length), and gating swing (d). The gating springs comprise tip links and other elements in series with the channels. To account for the elements inside the stereocilia that may contribute to the gating spring, we set the gating length equal to the tip-link length plus the radius of the stereocilia

$$x_i = x_{TL,i} + \frac{c}{2}, \tag{1}$$

in which $x_{TL,i}$ is the tip-link length.

The Lagrangian for the OHB is

$$L = -\left[\sum_{i=1}^{3} \frac{\kappa(\theta_i - \theta_u)^2}{2} + \sum_{i=1}^{2} \frac{k_{gs}(x_i - x_u - dP_i)^2}{2}\right], \tag{2}$$

in which the gating-spring length is $x_i - dP_i$. The variables θ_i and x_i depend on the displacement of the row-1 stereocilium (X_1). Thus, the steady-state equation for the OHB displacement X_1 in response to a constant stimulus force F_{ext} is given by

$$-\frac{\partial L}{\partial X_1} = F_{ext}. \tag{3}$$

We assume that neighboring stereocilia in different rows remain in sliding contact. Then the variables are given as explicit functions of the morphological parameters $\ell_1, \ell_2, \ell_3, c, a_1, a_2, b_1, b_2$, and the displacement X_1 as follows (ℓ_i is the height of the ith stereocilium, c is the stereocilium diameter (width), a_i is the distance between the pivots of stereocilium i and $i+1$, and b_i is the upper tip-link insertion distance for stereocilium i).

$$\theta_1(\ell_1, X_1) = \sin^{-1}\left[\frac{X_1}{\ell_1}\right], \tag{4}$$

$$\theta_2(a_1, c, \ell_1, \ell_2, X_1) = \theta_1(\ell_1, X_1) + \sin^{-1}\left[\left(\frac{a_1}{\ell_2}\right)\cos\theta_1(\ell_1, \ell_2, X_1) - \left(\frac{c}{\ell_2}\right)\right], \tag{5}$$

$$\theta_3(a_1, a_2, c, \ell_1, \ell_2, \ell_3, X_1) = \theta_2(a_1, c, \ell_1, \ell_2, X_1) + \sin^{-1}\left[\left(\frac{a_2}{\ell_3}\right)\cos\theta_2(a_1, c, \ell_1, \ell_2, X_1) - \left(\frac{c}{\ell_3}\right)\right], \tag{6}$$

$$x_1(a_1, b_1, c, \ell_1, \ell_2, X_1) = \sqrt{\frac{c^2}{4} + (b_1 - s_1(a_1, c, \ell_1, \ell_2, X_1))^2}, \text{ and} \tag{7}$$

$$x_2(a_1, a_2, b_2, c, \ell_1, \ell_2, \ell_3, X_1) = \sqrt{\frac{c^2}{4} + (b_2 - s_2(a_1, a_2, c, \ell_1, \ell_2, \ell_3, X_1))^2}, \tag{8}$$

in which

$$
\begin{aligned}
s_1(a_1, c, \ell_1, \ell_2, X_1) &= -a_1 \sin\theta_1(\ell_1, X_1) \\
&\quad + \ell_2 \cos\left(\theta_2(a_1, c, \ell_1, \ell_2, X_1) - \theta_1(\ell_1, X_1)\right) \text{and} \qquad (9) \\
s_2(a_1, a_2, c, \ell_1, \ell_2, \ell_3, X_1) &= -a_2 \sin\theta_2(a_1, c, \ell_1, \ell_2, X_1) \\
&\quad + \ell_3 \cos\left(\theta_3(a_1, a_2, c, \ell_1, \ell_2, \ell_3, X_1) - \theta_2(a_1, c, \ell_1, \ell_2, X_1)\right) \qquad (10)
\end{aligned}
$$

are the distances from the pivot points to the sliding contact point locations along the central axes of the stereocilia.

Using Eqs 3–8, the steady-state governing equation for the OHB is

$$
\begin{aligned}
F_{ext} = &\, \kappa(\theta_1(\ell_1, X_1) - \theta_u)\frac{d\theta_1(\ell_1, X_1)}{dX_1} + \kappa(\theta_2(a_1, c, \ell_1, \ell_2, X_1) - \theta_u)\frac{d\theta_2(a_1, c, \ell_1, \ell_2, X_1)}{dX_1} \\
&+ \kappa(\theta_3(a_1, a_2, c, \ell_1, \ell_2, \ell_3, X_1) - \theta_u)\frac{d\theta_3(a_1, a_2, c, \ell_1, \ell_2, \ell_3, X_1)}{dX_1} \\
&+ k_{gs}(x_1(a_1, b_1, c, \ell_1, \ell_2, X_1) - x_u - P_1 d)\frac{dx_1(a_1, b_1, c, \ell_1, \ell_2, X_1)}{dX_1} \\
&+ k_{gs}(x_2(a_1, a_2, b_2, c, \ell_1, \ell_2, \ell_3, X_1) - x_u - P_2 d)\frac{dx_2(a_1, a_2, b_2, c, \ell_1, \ell_2, \ell_3, X_1)}{dX_1}. \qquad (11)
\end{aligned}
$$

We assume stereocilia are perpendicular to the hair-cell apex in the absence of links or stimulus forces, i.e., $\theta_u = 0$.

Each mechanoelectrical-transduction channel is described by a two-state system, one open and one close state. The normalized currents P_i are then given by Boltzmann functions of

the form

$$P_1(a_1, b_1, c, \ell_1, \ell_2, X_1) = \left[1 + \exp\left(\frac{-k_{gs}d(x_1(a_1, b_1, c, \ell_1, \ell_2, X_1) - x_h)}{k_B T}\right)\right]^{-1} \quad \text{and} \quad (12)$$

$$P_2(a_1, a_2, b_2, c, \ell_1, \ell_2, \ell_3, X_1) = \left[1 + \exp\left(\frac{-k_{gs}d(x_2(a_1, a_2, b_2, c, \ell_1, \ell_2, \ell_3, X_1) - x_h)}{k_B T}\right)\right]^{-1}, \quad (13)$$

in which k_B is the Boltzmann constant and T is the absolute temperature (S1).

Constraints derived from physiological observations in low extracellular calcium

The available experimental data requires us to start the fitting procedure using morphological measurements in low extracellular calcium. However, OHB morphology is usually measured in high extracellular calcium. We approximate the morphology in low extracellular calcium using measurements in high extracellular calcium. Our results imply that external calcium only changes the row-2 and row-3 heights by about 10 nm, $< 1\%$ of a stereocilium's height. The heights, widths, and pivot separations of the stereocilia are the same as prior work and are based on experimental measurements in high extracellular calcium (Table 1) (S2). Experimental observations of the OHB's state enable us to constrain the remaining parameter values (Table 2).

Using Eqs 11, we derive mathematical constraints for the resting OHB displacement without gating springs (X_1^{**}), the resting OHB displacement with gating springs (X_{1E}^{*}), the pivot stiffness (κ), the upper tip-link insertion distances (b_1, b_2), and the half-activation length (x_h).

1. *Determining the resting OHB displacement without gating springs,* X_1^{**}: The mathematical constraint for the resting OHB without gating springs is derived by setting

$F_{ext} = 0$ and $k_{gs} = 0$ in Eq 11 yielding

$$\sum_{i}^{N_s} \theta_i(X_1^{**}) \left(\frac{d\theta_i(X_1)}{dX_1} \right)_{X_1 = X_1^{**}} = 0, \tag{14}$$

which we solve to find X_1^{**}.

2. *Determining the resting OHB displacement with gating springs,* X_{1E}^*: The resting OHB position has been measured to change by $\Delta_E = 45$ nm when the gating springs are disrupted (Table 2). Therefore,

$$X_{1E}^* = X_1^{**} - \Delta_E. \tag{15}$$

3. *Determining the pivot stiffness,* κ: The OHB's stiffness without gating springs (K_{HB}^{noGS}) has been measured (Table 2), implying that the stiffness of a column is K_{HB}^{noGS}/N_c, in which N_c is the number of columns (Table 1). We equate K_{HB}^{noGS}/N_c with the linearization of dF_{ext}/dX_1 at the resting position X_1^{**} from Eq 11 with $k_{gs} = 0$, yielding

$$\frac{K_{HB}^{noGS}}{N_c} = \kappa \left[\sum_{i=1}^{N_s} \left(\frac{d\theta_i(X_1)}{dX_1} \right)^2 + \theta_i(X_1) \frac{d^2\theta_i(X_1)}{dX_1^2} \right]_{X_1 = X_1^{**}}, \tag{16}$$

which we solve to find the pivot stiffness κ.

4. *Determining the upper tip-link insertion distances,* b_1, b_2: We assume that myosin motors attached to the gating springs set the gating lengths at rest to be equal in low calcium

$$x_{1E}^* = x_E^* \text{ and} \tag{17}$$

$$x_{2E}^* = x_E^*. \tag{18}$$

The resting length of the tip links has been measured to be $x^*_{TL} = 186$ nm (Table 2). Using Eq 1, we get

$$x^*_E = x^*_{TL} + \frac{c}{2}. \tag{19}$$

We determine the upper tip-link insertion distances b_1 and b_2 using geometry (Eqs 7, 8, 15, and 19; Fig S1), yielding

$$b_1 = \sqrt{x^{*2}_E - \frac{c^2}{4}} + s_1(a_1, c, \ell_1, \ell_2, X^*_{1E}) \text{ and} \tag{20}$$

$$b_2 = \sqrt{x^{*2}_E - \frac{c^2}{4}} + s_2(a_1, a_2, c, \ell_1, \ell_2, \ell_3, X^*_{1E}), \tag{21}$$

in which s_1 and s_2 are given by Eqs 9 and 10. In the model presented here, we do not allow b_1 and b_2 to change with extracellular calcium (changing b_1 and b_2 has been proposed to account for slow adaptation) (S3).

5. *Determining the half-activation length* x_h: We assume that the resting gating lengths are equal and know that the normalized receptor current has been measured to be 0.5 (Table 2). Additionally, the normalized receptor current equals the mean of the normalized channel currents. Therefore, the resting channel currents are each equal to 0.5. Using Eqs 12, 13, and 19, we find

$$x_h = x^*_E. \tag{22}$$

6. *Constraint using the resting displacement,* X^*_{1E}: The resting displacement X^*_{1E} is a

solution of Eq 11 when $F_{ext} = 0$, yielding

$$
\begin{aligned}
0 = \kappa & \left[\theta_1(X_1)\frac{d\theta_1(X_1)}{dX_1} + \theta_2(X_1)\frac{d\theta_2(X_1)}{dX_1} + \theta_3(X_1)\frac{d\theta_3(X_1)}{dX_1}\right]_{X_1=X_{1E}^*} \\
& + k_{gs}(x_1(X_{1E}^*) - x_u - dP_1(X_{1E}^*))\left(\frac{dx_1(X_1)}{dX_1}\right)_{X_1=X_{1E}^*} \\
& + k_{gs}(x_2(X_{1E}^*) - x_u - dP_2(X_{1E}^*))\left(\frac{dx_2(X_1)}{dX_1}\right)_{X_1=X_{1E}^*}.
\end{aligned}
\tag{23}
$$

We use this constraint in combination with the constraints described next.

Constraints derived from physiological observations in high extracellular calcium

We allow the heights of rows 2 and 3 (ℓ_{2P} and ℓ_{3P}) and the resting displacement (X_{1P}^*) in high calcium to be different than in low calcium (Tables 1 and 3). We predict the values of ℓ_{2P}, ℓ_{3P}, X_{1P}^*, the gating-spring stiffness k_{gs}, the gating-spring unloaded length x_u, and the gating swing d using Eq 23 and the following constraints.

1. Like the low-calcium case, we assume that the resting gating-spring lengths are the same in high calcium, implying that the resting channel currents are equal ($P_{1P}^* = P_{2P}^* = P_P^*$). The resting receptor current in high calcium has been measured to be 0.087, implying that $P_P^* = 0.087$ (Table 2). We thus have the following constraints

$$P_1(\ell_{2P}, X_{1P}^*) = P_P^* \text{ and} \tag{24}$$

$$P_2(\ell_{2P}, \ell_{3P}, X_{1P}^*) = P_P^*. \tag{25}$$

2. In the measured receptor-current activation curve, the distance between the curve's

center (at which the receptor-current equals 0.5) and the resting displacement is $X_J = 40$ nm (Table 2). This leads to the following constraint.

$$P_1(\ell_{2P}, X_{1P}^* + X_J) + P_2(\ell_{2P}, \ell_{3P}, X_{1P}^* + X_J) = 1. \tag{26}$$

3. At rest in high calcium, the resting governing equation is ($F_{ext} = 0$, $\ell_2 = \ell_{2P}$, and $\ell_3 = \ell_{3P}$ in Eq 11)

$$\begin{aligned}
0 = \kappa & \left[\theta_1(X_1)\frac{d\theta_1(X_1)}{dX_1} + \theta_2(\ell_{2P}, X_1)\frac{d\theta_2(\ell_{2P}, X_1)}{dX_1} \right. \\
& \left. + \theta_3(\ell_{2P}, \ell_{3P}, X_1)\frac{d\theta_3(\ell_{2P}, \ell_{3P}, X_1)}{dX_1}\right]_{X_1 = X_{1P}^*} \\
& + k_{gs}(x_1(\ell_{2P}, X_{1P}^*) - x_u - dP_1(\ell_{2P}, X_{1P}^*)) \left(\frac{dx_1(\ell_{2P}, X_1)}{dX_1}\right)_{X_1 = X_{1P}^*} \\
& + k_{gs}(x_2(\ell_{2P}, \ell_{3P}, X_{1P}^*) - x_u - dP_2(\ell_{2P}, \ell_{3P}, X_{1P}^*)) \left(\frac{dx_2(\ell_{2P}, \ell_{3P}, X_1)}{dX_1}\right)_{X_1 = X_{1P}^*}.
\end{aligned} \tag{27}$$

4. The OHB's stiffness (K_{HB}) has been measured in high calcium (Table 2), implying that the stiffness of a column is K_{HB}/N_c. We equate K_{HB}/N_c with the linearization

of dF_{ext}/dX_1 at the resting position X_{1P}^* from Eq 11, yielding

$$\begin{aligned}\frac{K_{HB}}{N_c} &= \kappa\left[\left(\frac{d\theta_1(X_1)}{dX_1}\right)^2+\left(\frac{d\theta_2(\ell_{2P},X_1)}{dX_1}\right)^2+\left(\frac{d\theta_3(\ell_{2P},\ell_{3P},X_1)}{dX_1}\right)^2\right]_{X_1=X_{1P}^*}\\
&+\kappa\left[\theta_1(X_1)\frac{d^2\theta_1(X_1)}{dX_1^2}+\theta_2(\ell_{2P},X_1)\frac{d^2\theta_2(\ell_{2P},X_1)}{dX_1^2}\right]_{X_1=X_{1P}^*}\\
&+\kappa\left[\theta_3(\ell_{2P},\ell_{3P},X_1)\frac{d^2\theta_3(\ell_{2P},\ell_{3P},X_1)}{dX_1^2}\right]_{X_1=X_{1P}^*}\\
&+k_{gs}\left[\left(\frac{dx_1(\ell_{2P},X_1)}{dX_1}\right)^2+\left(\frac{dx_2(\ell_{2P},\ell_{3P},X_1)}{dX_1}\right)^2\right]_{X_1=X_{1P}^*}\\
&+k_{gs}\left(x_1(\ell_{2P},X_{1P}^*)-x_u-dP_1(\ell_{2P},X_{1P}^*)\right)\\
&\left[\left(\frac{d^2x_1(\ell_{2P},X_1)}{dX_1^2}-k_{gs}d\frac{dP_1(\ell_{2P},X_1)}{dX_1}\right)\frac{dx_1(\ell_{2P},X_1)}{dX_1}\right]_{X_1=X_{1P}^*}\\
&+k_{gs}(x_2(\ell_{2P},\ell_{3P},X_{1P}^*)-x_u-dP_2(\ell_{2P},\ell_{3P},X_{1P}^*))\\
&\left[\left(\frac{d^2x_2(\ell_{2P},\ell_{3P},X_1)}{dX_1^2}-k_{gs}d\frac{dP_2(\ell_{2P},\ell_{3P},X_1)}{dX_1}\right)\frac{dx_2(\ell_{2P},\ell_{3P},X_1)}{dX_1}\right]_{X_1=X_{1P}^*}.\end{aligned} \tag{28}$$

We now have 6 constraints, Eqs 23–28, which we solve to find the 6 unknowns $\ell_{2P}, \ell_{3P}, X_{1P}^*, k_{gs}, d$ and x_u. The full list of unknowns and constraints is given in Table S1.

Unknown	Constraint
**Low calcium**	
κ	$\frac{K_{HB}^{noGS}}{N_c} = \kappa \left[\sum_{i=1}^{N_s} \left(\frac{d\theta_i(X_1)}{dX_1}\right)^2 + \theta_i(X_1)\frac{d^2\theta_i(X_1)}{dX_1^2}\right]_{X_1=X_1^{**}}$ (Eq 16)
b_1	$b_1 = \sqrt{x_{1E}^{*2} - \frac{c^2}{4}} + s_1$ (Eq 20)
b_2	$b_2 = \sqrt{x_{2E}^{*2} - \frac{c^2}{4}} + s_2$ (Eq 21)
x_h	$x_h = x_E^*$ (Eq 22)
x_u	$\dot{X}_{1E}^* = 0$ (Eq 23)
X_1^{**}	$\sum_i^{N_s} \theta_i(X_1)\frac{d\theta_i(X_1)}{dX_1} = 0$ (Eq 14)
X_{1E}^*	$X_{1E}^* = X_1^{**} - \Delta_E$ (Eq 15)
$x_1(X_{1E}^*)$	$x_1(X_{1E}^*) = x_E^*$ (Eq 17)
$x_2(X_{1E}^*)$	$x_2(X_{1E}^*) = x_E^*$ (Eq 18)
x_E^*	$x_E^* = x_{TL}^* + \frac{c}{2}$ (Eq 19)
**High calcium**	
ℓ_{2P}	$\left[1 + \exp\left(-\frac{k_{gs}d(x_1(\ell_{2P}, X_{1P}^*) - x_h)}{k_B T}\right)\right]^{-1} = P_P^*$ (Eq 24)
ℓ_{3P}	$\left[1 + \exp\left(\frac{-k_{gs}d(x_2(\ell_{2P}, \ell_{3P}, X_{1P}^*) - x_h)}{k_B T}\right)\right]^{-1} = P_P^*$ (Eq 25)
k_{gs}	$\frac{K_{HB}}{N_c} = \kappa \left[\left(\frac{d\theta_1(X_1)}{dX_1}\right)^2 + \left(\frac{d\theta_2(\ell_{2P}, X_1)}{dX_1}\right)^2 + \left(\frac{d\theta_3(\ell_{2P}, \ell_{3P}, X_1)}{dX_1}\right)^2\right]_{\mathrm{X_1=X_{1P}^*}}$ $+\kappa \left[\theta_1(X_1)\frac{d^2\theta_1(X_1)}{dX_1^2} + \theta_2(\ell_{2P}, X_1)\frac{d^2\theta_2(\ell_{2P}, X_1)}{dX_1^2} + \theta_3(\ell_{2P}, \ell_{3P}, X_1)\frac{d^2\theta_3(\ell_{2P}, \ell_{3P}, X_1)}{dX_1^2}\right]_{\mathrm{X_1=X_{1P}^*}}$ $+k_{gs}\left[\left(\frac{dx_1(\ell_{2P}, X_1)}{dX_1}\right)^2 + \left(\frac{dx_2(\ell_{2P}, \ell_{3P}, X_1)}{dX_1}\right)^2\right]_{\mathrm{X_1=X_{1P}^*}}$ $+k_{gs}\left(x_1(\ell_{2P}, X_{1P}^*) - x_u - dP_1(\ell_{2P}, X_{1P}^*)\right)\left[\left(\frac{d^2x_1(\ell_{2P}, X_1)}{dX_1^2} - k_{gs}d\frac{dP_1(\ell_{2P}, X_1)}{dX_1}\right)\frac{dx_1(\ell_{2P}, X_1)}{dX_1}\right]_{\mathrm{X_1=X_{1P}^*}}$ $+k_{gs}(x_2(\ell_{2P}, \ell_{3P}, X_{1P}^*) - x_u - dP_2(\ell_{2P}, \ell_{3P}, X_{1P}^*))$ $\left[\left(\frac{d^2x_2(\ell_{2P}, \ell_{3P}, X_1)}{dX_1^2} - k_{gs}d\frac{dP_2(\ell_{2P}, \ell_{3P}, X_1)}{dX_1}\right)\frac{dx_2(\ell_{2P}, \ell_{3P}, X_1)}{dX_1}\right]_{\mathrm{X_1=X_{1P}^*}}$ (Eq 28)
d	$\dot{X}_{1P}^* = 0$ (Eq 27)
X_{1P}^*	$\left[1 + \exp\left(\frac{-k_{gs}d(x_1(\ell_{2P}, X_{1P}^* + X_J) - x_h)}{k_B T}\right)\right]^{-1} + \left[1 + \exp\left(\frac{-k_{gs}d(x_2(\ell_{2P}, \ell_{3P}, X_{1P}^* + X_J) - x_h)}{k_B T}\right)\right]^{-1} = 1$ (Eq 26)
$x_1(\ell_{2P}, X_{1P}^*)$	$x_1(\ell_{2P}, X_{1P}^*) = \sqrt{R^2 + (b_1 - s_1(\ell_{2P}, X_{1P}^*))^2}$ (Eq 7)
$x_2(\ell_{2P}, \ell_{3P}, X_{1P}^*)$	$x_2(\ell_{2P}, \ell_{3P}, X_{1P}^*) = \sqrt{R^2 + (b_2 - s_2(\ell_{2P}, \ell_{3P}, X_{1P}^*))^2}$ (Eq 8)

Table S1: The number of unknowns equals the number of constraints, enabling us to calculate all the parameter values and variables' resting-state values in low and high calcium.

Unknown	Prediction	Unknown	Prediction
**Low calcium**		**High calcium**	
**Parameter**	**Value**	**Parameter**	**Value**
κ	0.7 fN.m/rad	ℓ_{2P}	2111.7 nm
b_1	2491.3 nm	ℓ_{3P}	1311.5 nm
b_2	1590.7 nm	k_{gs}	2.1 mN/m
x_h	330.5 nm	d	0.7 nm
x_u	316.9 nm		
**Variable**	**Value**	**Variable**	**Value**
X_1^{**}	−626.8 nm	X_{1P}^*	−645.9 nm
X_{1E}^*	−671.8 nm	$x_1(\ell_{2P}, X_{1P}^*)$	316.9 nm
$x_1(X_{1E}^*)$	330.5 nm	$x_2(\ell_{2P}, \ell_{3P}, X_{1P}^*)$	323.9 nm
$x_2(X_{1E}^*)$	330.5 nm		
x_E^*	330.5 nm		

Table S2: Predicted parameter values and resting-state variable values for the default morphology and the minimum value for $\ell_1 = \text{mean} - \text{standard deviation} = 3468$ nm (Table 1). The parameters are the upper tip-link insertion distances for row 1 and row 2 (b_1 and b_2), the stiffness of the stereocilium pivots (κ), the stiffness (k_{gs}) and the unloaded length (x_u) of the gating springs, the gating swing of the channels (d), the half-activation length of the gating springs (x_h), and the heights of row 2 and row 3 in high calcium (ℓ_{2P} and ℓ_{3P}). The resting-state variables are the resting OHB displacement without gating springs (X_1^{**}), the resting OHB displacement in low calcium (X_{1E}^*), the resting gating lengths in low calcium ($x_1(X_{1E}^*)$ and $x_2(X_{1E}^*)$), the resting tip-link length plus the stereocilium radius in low calcium (x_E^*), the resting OHB displacement in high calcium (X_{1P}^*), and the resting gating lengths in high calcium ($x_1(\ell_{2P}, X_{1P}^*)$ and $x_2(\ell_{2P}, \ell_{3P}, X_{1P}^*)$).

Unknown	Prediction	Unknown	Prediction
**Low calcium**		**High calcium**	
**Parameter**	**Value**	**Parameter**	**Value**
κ	1.4 fN.m/rad	ℓ_{2P}	2108.6 nm
b_1	2489.2 nm	ℓ_{3P}	1308.4 nm
b_2	1588.3 nm	k_{gs}	3.8 mN/m
x_h	330.5 nm	d	0.5 nm
x_u	320.6 nm		
**Variable**	**Value**	**Variable**	**Value**
X_1^{**}	−855.2 nm	X_{1P}^*	−874.0 nm
X_{1E}^*	−900.2 nm	$x_1(\ell_{2P}, X_{1P}^*)$	325.7 nm
$x_1(X_{1E}^*)$	330.5 nm	$x_2(\ell_{2P}, \ell_{3P}, X_{1P}^*)$	325.7 nm
$x_2(X_{1E}^*)$	330.5 nm		
x_E^*	330.5 nm		

Table S3: Predicted parameter values and resting-state variable values for the default morphology and the maximum value for ℓ_1 = mean + standard deviation = 4732 nm (Table 1). The parameters are the upper tip-link insertion distances for row 1 and row 2 (b_1 and b_2), the stiffness of the stereocilium pivots (κ), the stiffness (k_{gs}) and the unloaded length (x_u) of the gating springs, the gating swing of the channels (d), the half-activation length of the gating springs (x_h), and the heights of row 2 and row 3 in high calcium (ℓ_{2P} and ℓ_{3P}). The resting-state variables are the resting OHB displacement without gating springs (X_1^{**}), the resting OHB displacement in low calcium (X_{1E}^*), the resting gating lengths in low calcium ($x_1(X_{1E}^*)$ and $x_2(X_{1E}^*)$), the resting tip-link length plus the stereocilium radius in low calcium (x_E^*), the resting OHB displacement in high calcium (X_{1P}^*), and the resting gating lengths in high calcium ($x_1(\ell_{2P}, X_{1P}^*)$ and $x_2(\ell_{2P}, \ell_{3P}, X_{1P}^*)$).

**Unknown**	**Prediction**	**Unknown**	**Prediction**
**Low calcium**		**High calcium**	
**Parameter**	**Value**	**Parameter**	**Value**
κ	1.0 fN.m/rad	ℓ_{2P}	1640.7 nm
b_1	2171.8 nm	ℓ_{3P}	1309.9 nm
b_2	1585.0 nm	k_{gs}	2.8 mN/m
x_h	330.5 nm	d	0.6 nm
x_u	318.9 nm		
**Variable**	**Value**	**Variable**	**Value**
X_1^{**}	−810.7 nm	X_{1P}^*	−828.9 nm
X_{1E}^*	−855.7 nm	$x_1(\ell_{2P}, X_{1P}^*)$	324.9 nm
$x_1(X_{1E}^*)$	330.5 nm	$x_2(\ell_{2P}, \ell_{3P}, X_{1P}^*)$	324.9 nm
$x_2(X_{1E}^*)$	330.5 nm		
x_E^*	330.5 nm		

Table S4: Predicted parameter values and resting-state variable values for the default morphology and the minimum value for $\ell_2 = 1775$ nm (Table 1). The geometry requires that $b_2 \leq \ell_2 - c/2$ and $b_2 \geq \ell_3 + x_{tl}^*$. To satisfy the geometric requirement, we choose $\ell_2 = \ell_3 + x_{tl}^* + c = 1775$ nm as the minimum value for ℓ_2 consistent with the experimental data (Table 1). The parameters are the upper tip-link insertion distances for row 1 and row 2 (b_1 and b_2), the stiffness of the stereocilium pivots (κ), the stiffness (k_{gs}) and the unloaded length (x_u) of the gating springs, the gating swing of the channels (d), the half-activation length of the gating springs (x_h), and the heights of row 2 and row 3 in high calcium (ℓ_{2P} and ℓ_{3P}). The resting-state variables are the resting OHB displacement without gating springs (X_1^{**}), the resting OHB displacement in low calcium (X_{1E}^*), the resting gating lengths in low calcium ($x_1(X_{1E}^*)$ and $x_2(X_{1E}^*)$), the resting tip-link length plus the stereocilium radius in low calcium (x_E^*), the resting OHB displacement in high calcium (X_{1P}^*), and the resting gating lengths in high calcium ($x_1(\ell_{2P}, X_{1P}^*)$ and $x_2(\ell_{2P}, \ell_{3P}, X_{1P}^*)$).

Unknown	Prediction	Unknown	Prediction
**Low calcium**		**High calcium**	
**Parameter**	**Value**	**Parameter**	**Value**
κ	1.1 fN.m/rad	ℓ_{2P}	2809.8 nm
b_1	3181.1 nm	ℓ_{3P}	1309.6 nm
b_2	1595.6 nm	k_{gs}	3.0 mN/m
x_h	330.5 nm	d	0.6 nm
x_u	319.3 nm		
**Variable**	**Value**	**Variable**	**Value**
X_1^{**}	−645.5 nm	X_{1P}^*	−665.1 nm
X_{1E}^*	−690.5 nm	$x_1(\ell_{2P}, X_{1P}^*)$	325.0 nm
$x_1(X_{1E}^*)$	330.5 nm	$x_2(\ell_{2P}, \ell_{3P}, X_{1P}^*)$	325.0 nm
$x_2(X_{1E}^*)$	330.5 nm		
x_E^*	330.5 nm		

Table S5: Predicted parameter values and resting-state variable values for the default morphology and the maximum value for ℓ_2 = mean + standard deviation = 2800 nm (Table 1). The parameters are the upper tip-link insertion distances for row 1 and row 2 (b_1 and b_2), the stiffness of the stereocilium pivots (κ), the stiffness (k_{gs}) and the unloaded length (x_u) of the gating springs, the gating swing of the channels (d), the half-activation length of the gating springs (x_h), and the heights of row 2 and row 3 in high calcium (ℓ_{2P} and ℓ_{3P}). The resting-state variables are the resting OHB displacement without gating springs (X_1^{**}), the resting OHB displacement in low calcium (X_{1E}^*), the resting gating lengths in low calcium ($x_1(X_{1E}^*)$ and $x_2(X_{1E}^*)$), the resting tip-link length plus the stereocilium radius in low calcium (x_E^*), the resting OHB displacement in high calcium (X_{1P}^*), and the resting gating lengths in high calcium ($x_1(\ell_{2P}, X_{1P}^*)$ and $x_2(\ell_{2P}, \ell_{3P}, X_{1P}^*)$).

Unknown	Prediction	Unknown	Prediction
**Low calcium**		**High calcium**	
**Parameter**	**Value**	**Parameter**	**Value**
κ	1.0 fN.m/rad	ℓ_{2P}	2110.7 nm
b_1	2515.7 nm	ℓ_{3P}	910.3 nm
b_2	1198.6 nm	k_{gs}	2.7 mN/m
x_h	330.5 nm	d	0.6 nm
x_u	319.2 nm		
**Variable**	**Value**	**Variable**	**Value**
X_1^{**}	−914.3 nm	X_{1P}^*	−929.3 nm
X_{1E}^*	−959.3 nm	$x_1(\ell_{2P}, X_{1P}^*)$	324.8 nm
$x_1(X_{1E}^*)$	330.5 nm	$x_2(\ell_{2P}, \ell_{3P}, X_{1P}^*)$	324.8 nm
$x_2(X_{1E}^*)$	330.5 nm		
x_E^*	330.5 nm		

Table S6: Predicted parameter values and resting-state variable values for the default morphology and the minimum value for ℓ_3 = mean − standard deviation = 900 nm (Table 1). The parameters are the upper tip-link insertion distances for row 1 and row 2 (b_1 and b_2), the stiffness of the stereocilium pivots (κ), the stiffness (k_{gs}) and the unloaded length (x_u) of the gating springs, the gating swing of the channels (d), the half-activation length of the gating springs (x_h), and the heights of row 2 and row 3 in high calcium (ℓ_{2P} and ℓ_{3P}). The resting-state variables are the resting OHB displacement without gating springs (X_1^{**}), the resting OHB displacement in low calcium (X_{1E}^*), the resting gating lengths in low calcium ($x_1(X_{1E}^*)$ and $x_2(X_{1E}^*)$), the resting tip-link length plus the stereocilium radius in low calcium (x_E^*), the resting OHB displacement in high calcium (X_{1P}^*), and the resting gating lengths in high calcium ($x_1(\ell_{2P}, X_{1P}^*)$ and $x_2(\ell_{2P}, \ell_{3P}, X_{1P}^*)$).

**Unknown**	**Prediction**	**Unknown**	**Prediction**
**Low calcium**		**High calcium**	
**Parameter**	**Value**	**Parameter**	**Value**
κ	1.0 fN.m/rad	ℓ_{2P}	2109.7 nm
b_1	2479.3 nm	ℓ_{3P}	1634.6 nm
b_2	1911.0 nm	k_{gs}	2.9 mN/m
x_h	330.5 nm	d	0.6 nm
x_u	319.2 nm		
**Variable**	**Value**	**Variable**	**Value**
X_1^{**}	−667.8 nm	X_{1P}^*	−687.7 nm
X_{1E}^*	−712.8 nm	$x_1(\ell_{2P}, X_{1P}^*)$	325.0 nm
$x_1(X_{1E}^*)$	330.5 nm	$x_2(\ell_{2P}, \ell_{3P}, X_{1P}^*)$	325.0 nm
$x_2(X_{1E}^*)$	330.5 nm		
x_E^*	330.5 nm		

Table S7: Predicted parameter values and resting-state variable values for the default morphology and the maximum value for $\ell_3 = 1625$ nm (Table 1). The geometry requires that $b_2 \le \ell_2 - c/2$ and $b_2 \ge \ell_3 + x_{tl}^*$. To satisfy the geometric requirement, we choose $\ell_3 = \ell_2 - x_{tl}^* - c = 1625$ nm as the maximum value for ℓ_3 consistent with the experimental data (Table 1). The parameters are the upper tip-link insertion distances for row 1 and row 2 (b_1 and b_2), the stiffness of the stereocilium pivots (κ), the stiffness (k_{gs}) and the unloaded length (x_u) of the gating springs, the gating swing of the channels (d), the half-activation length of the gating springs (x_h), and the heights of row 2 and row 3 in high calcium (ℓ_{2P} and ℓ_{3P}). The resting-state variables are the resting OHB displacement without gating springs (X_1^{**}), the resting OHB displacement in low calcium (X_{1E}^*), the resting gating lengths in low calcium ($x_1(X_{1E}^*)$ and $x_2(X_{1E}^*)$), the resting tip-link length plus the stereocilium radius in low calcium (x_E^*), the resting OHB displacement in high calcium (X_{1P}^*), and the resting gating lengths in high calcium ($x_1(\ell_{2P}, X_{1P}^*)$ and $x_2(\ell_{2P}, \ell_{3P}, X_{1P}^*)$).

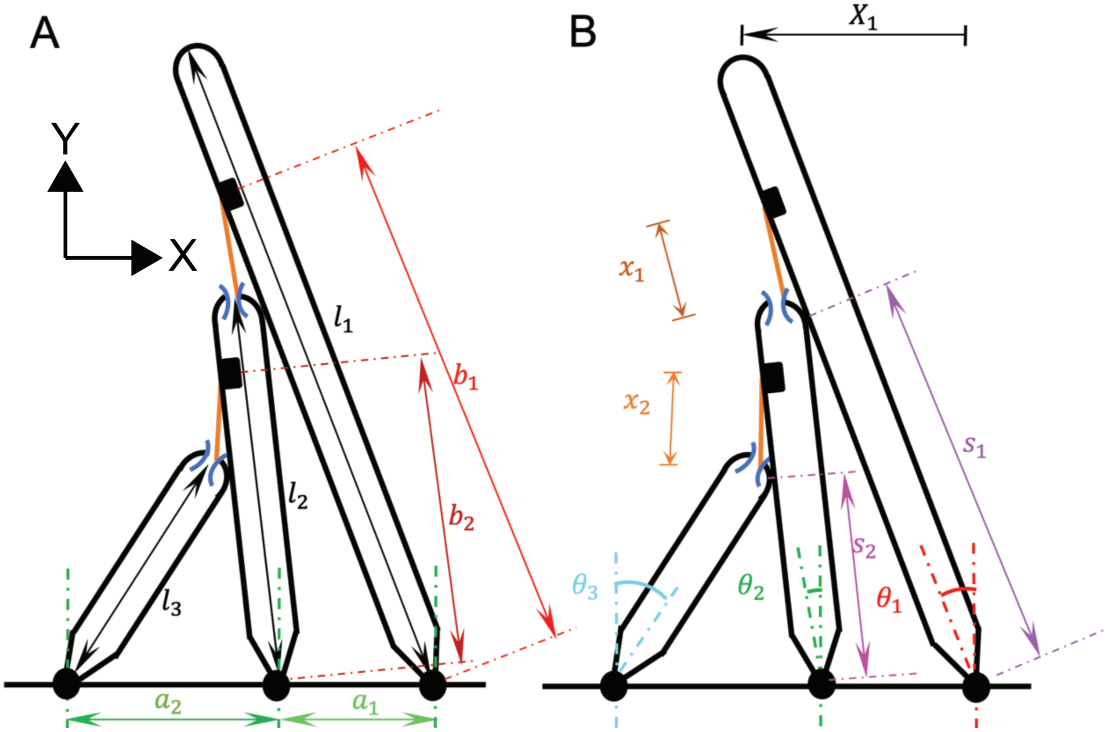

Fig S1. OHB morphology and state variables. (A) A schematic representing the OHB is shown with its morphological parameters labeled. OHB morphology is described by the heights of row 1, row 2 and row 3 (ℓ_1, ℓ_2, and ℓ_3; black), the pivot spacings between row 1 and row 2 (a_1; green), and between row 2 and row 3 (a_2; dark green), and the upper tip-link insertion distance for row 1 (b_1; red) and for row 2 (b_2; dark red). (B) A schematic representing the OHB is shown with its state variables labeled. The state of the OHB is described by the displacement of row 1 from the vertical (X_1; black), the gating lengths (x_1; dark brown, x_2; light orange), the contact position between stereocilia in rows 1 and 2 (s_1; violet), and in rows 2 and 3 (s_2; pink), and the angular displacements from the vertical for rows 1 (θ_1; red), 2 (θ_2; green) and 3 (θ_3; light blue).

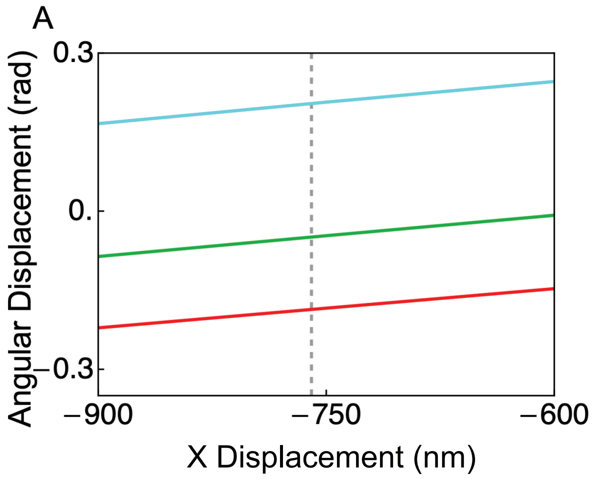

Fig S2. Within the physiological range, angular displacements increase linearly with OHB displacement. Predicted angular displacements from the hair-cell apex perpendicular are shown versus the OHB displacement for the stereocilia in row 1 (red), row 2 (green), and row 3 (light blue) in high (≥ 0.5 mM) (A) and low (≤ 50 μM) (B) extracellular calcium. Vertical dashed grey lines indicate the OHB displacements at rest.

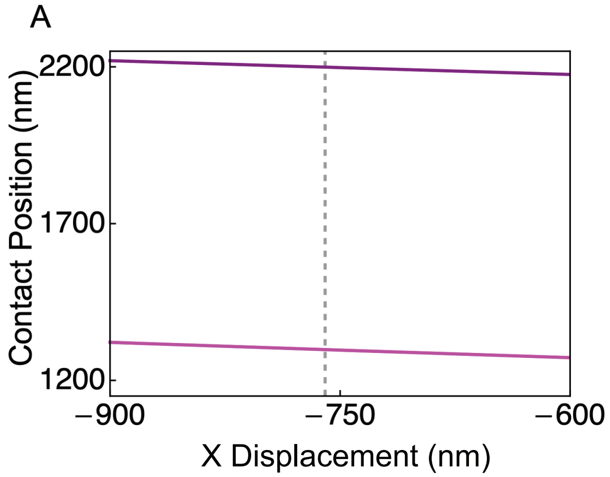

Fig S3. Within the physiological range, contact positions between stereocilia decrease linearly with OHB displacement. Predicted contact positions between stereocilia in rows 1 and 2 (violet) and in rows 2 and 3 (pink) are shown versus the OHB displacement in high (≥ 0.5 mM) (A) and low (≤ 50 μM) (B) extracellular calcium. Vertical dashed grey lines indicate the OHB displacements at rest.

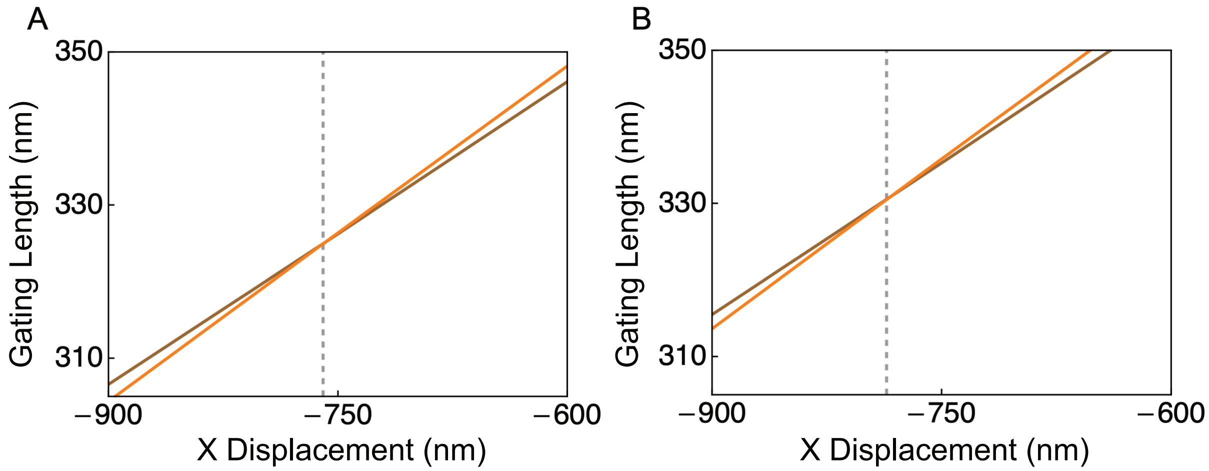

Fig S4. Within the physiological range, gating lengths increase linearly with OHB displacement. Predicted gating lengths for rows 1 (dark brown) and 2 (light orange) are shown versus the OHB displacement in high (≥ 0.5 mM) (A) and low ($\leq 50\ \mu$M) extracellular calcium (B). Vertical dashed grey lines indicate the OHB displacements at rest.

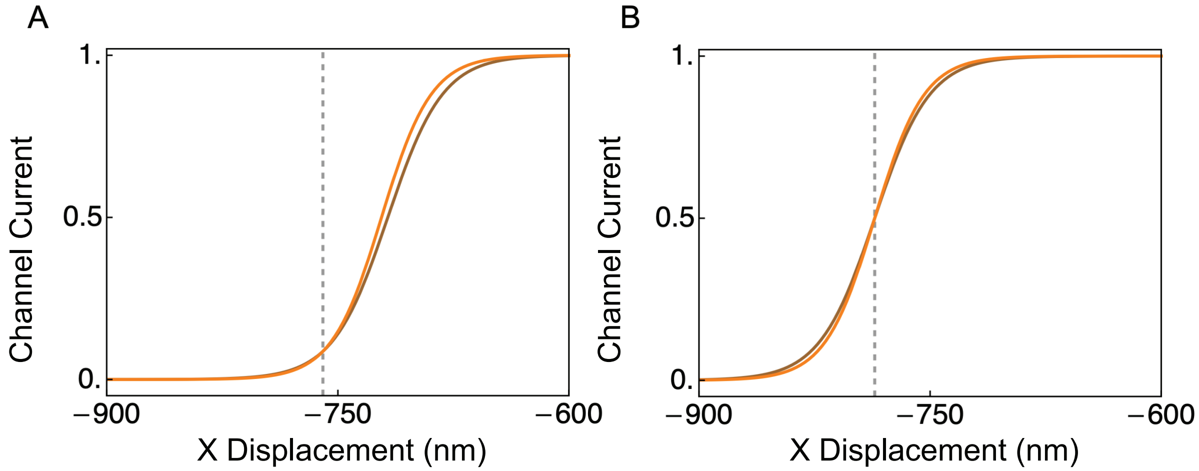

Fig S5. Row 1 and 2 channel currents differ little. Predicted currents for rows 2 (dark brown) and 3 (light orange) are shown versus the OHB displacement in high (≥ 0.5 mM) (A) and low ($\leq 50\ \mu$M) (B) extracellular calcium. Vertical dashed grey lines indicate the OHB displacements at rest.

Supporting References

S1 Corey DP, Hudspeth AJ (1983) Kinetics of the receptor current in bullfrog saccular hair cells. *J. Neuosci.* 3:962–976.

S2 Zhu Z, Reid W, George SS, Ou V, Ó Maoiléidigh D (2024) 3D morphology of an outer-hair-cell hair bundle increases its displacement and dynamic range. *Biophysical Journal.*

S3 Howard J, Hudspeth AJ (1987) Mechanical relaxation of the hair bundle mediates adaptation in mechanoelectrical transduction by the bullfrog's saccular hair cell. *Proc. Natl. Acad. Sci. USA* 84:3064–3068.
